# Bullying Perpetration, Moral Disengagement and Need for Popularity: Examining Reciprocal Associations in Adolescence

**DOI:** 10.1007/s10964-021-01482-4

**Published:** 2021-07-31

**Authors:** Eva M. Romera, Rosario Ortega-Ruiz, Kevin Runions, Antonio Camacho

**Affiliations:** 1 Universidad de Córdoba, Cordoba, Spain; 2 The University of Western Australia, Perth, Australia

**Keywords:** Motivation, Moral, Bullying, Between-person, Within-person, Longitudinal

## Abstract

Precursors and consequences of bullying have been widely explored, but much remains unclear about the association of moral and motivational factors. This study examined longitudinal associations between need for popularity, moral disengagement, and bullying perpetration. A total of 3017 participants, aged 11 to 16 years in wave 1 (49% girls; *M*_age_ = 13.15, *SD* = 1.09), were surveyed across four waves with six-month intervals. At the between-person level, cross-lagged modeling revealed a positive bidirectional association between moral disengagement and need for popularity; bullying perpetration was predicted by both need for popularity and moral disengagement. From the within-person level, random intercept cross-lagged analyses revealed that need for popularity predicted both moral disengagement and bullying perpetration. The results highlight the interplay between motivational and moral mechanisms that underlies bullying behavior.

## Introduction

Bullying is defined as intentional and repeated aggression characterized by an imbalance of power between perpetrator(s) and victim (Smith, 2019). Given bullying behavior significantly decreases during late school years (Cho & Lee, 2020), early and middle adolescence are hence target periods for effective intervention to prevent and reduce bullying. In recent years, important insights have arisen from studies on the psychological enablers of face-to-face bullying perpetration. Two important perspectives, in particular, have received empirical support. First, *moral disengagement* is posited as a set of psychological processes that may result in bullying by reducing self-censure when one is violating one’s moral standards (Bandura et al., 1996). Meta-analysis has confirmed a robust relationship of moral disengagment and bullying behavior (Gini et al., 2014). Second, an individual’s *need for popularity* is a foundational motivator in resource control theories of bullying. As postulated by such accounts, bullying is a strategic enactment of coercive manipulation, which may be used alongside self-interested pseudo-prosocial strategies to attain desired social resources, for example social dominance within one’s group (Clark et al., 2020). To date, the majority of studies have been cross-sectional, and the developmental sequence of the two constructs for adolescent bullying perpetration has been little studied. The current study used a longitudinal design over four waves to examine the developmental dynamics of moral disengagement, the need for popularity and face-to-face bullying perpetration over adolescence. In addition, gender and age were controlled (Smith et al., 2019).

### Bullying Perpetration and Moral Disengagement

Bandura’s social cognitive theory postulates that people establish moral standards as a self-regulatory process, to avoid self-censuring emotions like shame, remorse, and guilt (Bandura, 2002). In some cases, however, people may selectively and preemptively deactivate these emotional responses via moral disengagement, thereby enabling immoral behaviors (Bandura, 2002). Deactivation allows perpetrators to consider aggressive acts as appropriate and legitimate in view of their self-interest and it enables a positive self-image despite the immoral acts (Sticca & Perren, 2015). There is a large body of cross-sectional research that has focused on moral disengagement as a self-regulatory mechanism to explain peer aggression and bullying (see meta-analysis; Gini et al., 2014). Only a few studies have established longitudinal associations, and these showed that moral disengagement predicted future bullying perpetration after controlling for the autoregressive effects of perpetration (Falla et al., 2020; Visconti et al., 2015; Wang et al., 2017).

Social cognitive theory states that moral disengagement and immoral behavior reciprocate over time (Bandura et al., 1996), creating in effect a slippery slope to immorality. According to such a ‘breaking bad’ hypothesis, an instance of a morals-violating behavior is retroactively rationalized by moral disengagement, creating a bidirectional feedback loop toward increased likelihood of future bad behavior. Research findings are mixed. In a study of adolescents that examined whether bullying perpetration was prospectively associated with moral disengagement (Wang et al., 2017), the best fitting model included only a significant path from moral disengagement to subsequent bullying. Another article, however, used a standard cross-lagged panel model examining between-person associations over time, conducted with children (in 4th–6th grade), found that aggression predicted later moral disengagement (Visconti et al., 2015). A study during adolescence (in 7th–10th grade) has also found that involvement in aggression may lead to higher levels of moral disengagement later (Teng et al., 2019). This suggests that the deactivation of standard morals not only occurs before the aggression but may also arise from the use of aggression, as the progressive disengagement Bandura hypothesized (1996). More consequently, most studies about the bidirectionality of moral disengagement and aggression relied on traditional cross-lagged panel analyses; such models have been critiqued as considering only the between-person effects but failing to account adequately for within-person variance (see below).

### Bullying Perpetration and Need for Popularity

Adolescents are, generally, highly aware of their social position in the peer group and tend to desire increased visibility, influence, and power among their peers (Prinstein, 2018). Popular adolescents are perceived as prestigious, visible, and influential in the group’s decision making (Hogg, 2005). Popularity motivations may increase in importance relative to other social motivations during adolescence (Dawes & Xie, 2017). Socio-affective processing research has suggested that pubertal development may lead to increases in goals associated with achieving status and dominance (Meisel et al., 2021). Reflecting evolutionary-based group dynamics and adolescents’ need for popularity, status goals may be considered normative in adolescence (Moffitt, 1993). Researchers have assessed the need for popularity as a motivation to behave in ways that will be perceived as popular within a peer group (Santor et al., 2000). These developmental changes may make the need for popularity an increasingly important behavioral driver in adolescence.

Popularity motivations have been found to be precursors to a range of antisocial or problematic adolescent outcomes, including maladaptive social media use (i.e., sexting, mobile porn use or sexual grooming; Vanden Abeele et al., 2014; Utz et al., 2012). Longitudinal research indicated increased risk of unhealthy behaviors including alcohol use amongst those with high need for popularity (Malamut et al., 2020). The drive for popularity may motivate a range of risky behaviors among adolescents.

According to resource-control theory, individuals may use both pseudo-prosocial and aggressively coercive strategies to access and secure social resources, with the goal of attaining a position of social dominance (Hawley., 1999). In this approach, bullying is viewed as a deliberate strategy within the peer group to obtain the scarce resource of popularity and its perceived benefits (Huitsing et al., 2014). A range of studies have shown that popularity motivations are related to physical and social aggression towards peers (Dawes & Xie, 2014). Adolescents who report high use of resource-control strategies have a very high need for recognition from others and are the most involved in bullying perpetration (Clark et al., 2020). Some adolescents use coercive methods to elevate themselves in the social hierarchy (Ojanen & Nostrand, 2014), and adolescent bullying is associated with intentional reward motives for aggression (Dumas et al., 2019; Runions et al., 2018). Adolescents who are motivated to be popular will pay special attention to the potential benefits and cost of bullying behavior (Pouwels et al., 2019). If bullying appears to lead to popularity with minimal costs, young people are more likely to involve in bullying (Pan et al., 2020).

The developmental unfolding of bullying and the need for popularity has been little examined. The inverse influence is possible: social-cognitive theory (Bandura, 2002) would suggest that the reward value of successful instances of aggression (from the aggressor’s perspective) might increase the aggressor’s sense of agency, and could thereby feed the need for popularity. But bidirectionality between need for popularity and aggressive behavior has been tested in only a handful of studies. Previous research with early adolescents found that aggression predicts peer-nominated popularity in early adolescence (Stevens et al., 2020), suggesting it may be a successful strategy in some contexts. More tellingly, a two-wave study with Canadian students in grades 9–11 found that social aggression predicted significant increases in *need* for popularity assessed five months later (Dumas et al., 2019). However, a three-year prospective study found no such relationship between popularity goals and overall aggression, nor did popularity goals predict increased relational aggression (Malamut et al., 2020). To date, these studies have not examined bullying per se. Moreover, most studies analyzing the prospective associations between need for popularity and aggression used traditional cross-lagged panel analyses. Finally, these studies have not examined moral disengagement, which could entirely account for the relationship of need for popularity and bullying perpetration, as discussed next.

### Moral Disengagement and Need for Popularity

Although the need for popularity may motivate aggression, the literature is relatively silent on how young people manage to actually bring themselves to enact aggressive behavior in pursuit of popularity. Social-cognitive theory does, however, provide a theoretical account for the role of personal motivations in the progressive reduction of self-censure central to moral disengagement. For some adolescents, the moral justification of securing one’s position in the social group may provide a strong incentive for adolescents, especially in social settings where salient examples of aggression being rewarded by social status abound. Seeking social status for adolescents may be seen as a worthy pursuit, enabling ample self-serving moral justification (a key mechanism of moral disengagement). Bandura’s (2002) social cognitive theory posits that individual morality is rooted in one’s self-evaluations. Individuals with stronger need for popularity may be more strongly driven to disengage from the moral rules prohibiting them from reaching their goal. The attainment of the desired reward—social status—may allow individuals to disengage their moral controls by easily providing justifications to engage in immoral behavior. Moral disengagement, then, may provide a way to maintain a positive self-view while pursuing one’s goals through antisocial means.

Despite the theoretical link between motivation and moral functioning, few studies have explored this association. Adult empirical studies have found that high-status individuals are more likely to make immoral decisions because they more highly value their own welfare over that of other people (Piff et al., 2012). Also, the desire to obtain power is associated with impoverished understanding of others, and with insensitivity to social implications of behavior, with power being “associated with a reduced tendency to comprehend how other individuals see the world, think about the world, and feel about the world” (Galinsky et al., 2006, p. 1072). This suggests that attained power may faciliate moral disengagement via reduced perspective taking, but it does not speak directly to the need for popularity or related motivations to hold power.

During adolescence, popularity motivations drive an emphasis on anti-authoritarian behaviors associated with deviance from ethical standards, such as skipping school and damaging property (Dumas et al., 2019). But it remains unclear whether holding motives such as the need for popularity predispose youth to increased moral disengaagement. Amongst adolescents, higher levels of revenge goals have been found to predict greater moral disengagement (Visconti et al., 2015). The motivation to lead, referred to an individual’s desire or willingness to lead others is also associated with moral disengagement (Hinrichs et al., 2012). To date, however, no studies have examined whether the need for popularity predicts increases in moral disengagement over time, or whether the converse is the case.

### Between- and Within-Person Level Approach

Although moral disengagement and need for popularity have been mainly addressed as trait-like characteristics at between-person level, established over adolescence (Paciello et al., 2008; Dawes & Xie, 2017), both may also have state-like qualities at a within-person level. Moral disengagement implies a process of applying moral disengagement *mechanisms* (Bandura, 2018), and it has been argued that moral disengagement should not be considered only as a relatively stable characteristic of a person, but also as a process that assumes that an individual may exhibit differences in the expression of moral disengagement depending on the behavior, situation or context (Runions & Bak, 2015; Schaefer & Bouwmeester, 2020). This state-like characteristic of moral disengagement permits an understanding of how morally healthy individuals may engage in unethical behavior. Consequently, some have argued that study designs should adapt to the conceptualization of moral disengagement as a disposition and as a process (Moore, 2015). In this line, research has captured short-term intrapersonal changes in moral disengagement at six-month intervals (Sticca & Parren, 2015) and has even developed changes on its relationship with aggression at different points during adolescence at the within-person level (Teng et al., 2019). Need for popularity is also considered both a trait- and state-like variable (McDonald & Asher, 2018), and has also been considered to be malleable during socialization in adolescence (Makara & Madjar, 2015). Thus far, however, studies have measured social motivations as broad traits. However, in short-term studies, personal and contextual factors were found to be relevant in explaining changes in status goals (Makara & Madjar, 2015; Ojanen & Findley-Van Nostrand, 2020). In the case of bullying behavior, most research assumes methods that imply an absence of ﻿time-invariant individual differences. This appears to be a rather challenging statement, as involvement in perpetration among adolescents is highly variant: while some individuals are chronic bullies, others may be involved occasionally or even participate in different bullying behaviors (Zych et al., 2020). To address these gaps, recent short-term prospective research has already accounted for the inclusion of random intercepts in modeling adolescents bullying participation (Doty et al., 2020). Application of statistical methods that can adequately account for both state- (time-dependent characteristics) or trait-like variables (time-independent characteristics), such as random intercept cross-lagged model (RI-CLPM), is crucial to the study of potential drivers of bullying.

Recent methodological advances in cross-lagged panel models have provided improved ways to study of the association between variables from an individual-developmental perspective. The approach used in traditional cross-lagged panel model (CLPM) of simple autocorrelation—which accounts for rank-stability over time – fails to capture stability of trait-like variables within participants (Hamaker et al., 2015). However, both models have different properties and advantages and give useful information to understand the relationship between psychological variables (Hudson et al., 2019). At the between-person level, it may be useful to know how adolescents are situated within the group and which individuals are more likely to have higher or lower levels of psychological variables than their peers. Within-person modeling should be considered to explore the development at the individual level (Hudson et al., 2019). The present study adopts both approaches to understand the nature of the longitudinal associations between the study variables at the between- and within-person levels.

## Current Study

The present study provides a test of the prospective unfolding of bullying perpetration, moral disengagement and the need for popularity within adolescents. By using CLPM and RI-CLPM, this study brings to bear important developments in testing the relationships across these variables both the between- and within-person approach. Based on theoretical considerations it was hypothesized that bullying perpetration and moral disengagement would predict one another bidirectionally (Hypothesis 1). It was also predicted that need for popularity would predict both subsequent bullying (Hypothesis 2) and subsequent moral disengagement (Hypothesis 3). Gender and age were treated as control variables because both boys and middle adolescents have consistently reported higher perpetration involvement than girls and early adolescents (Smith et al., 2019).

## Methods

### Participants and Procedure

Participants were Spanish adolescents from a longitudinal study of risk and protective factors for bullying. A total of 3017 adolescents (49% girls) between 11 and 16 years participated from Grade 7 (*n* = 1,050; 46% girls; *M*_ageW1_ = 12.14, *SD* = 0.64), Grade 8 (*n* = 1,027; 50% girls; *M*_ageW1_ = 13.19, *SD* = 0.70) and Grade 9 (*n* = 940; 53% girls; *M*_ageW1_ = 14.23, *SD* = 0.70), recruited from 115 classes at 13 middle schools. Data were collected over 18 months in 4 waves, 6 months apart. Ethical approval for the study was provided by the research ethics committee of the corresponding author’s institution. After approval from the heads of school to participate in the study, permission from the regional government and active participants’ parents were obtained. All data were collected in the students’ home classroom during regular school hours. For each adolescent, a unique code based on their name and date of birth was generated and used to link the four data collection waves. Data were collected by trained and experienced interviewers. Adolescents received standardized instructions in which they were assured that no answers were right or wrong and that their participation in the study was confidential and voluntary, and that they could leave the study at any time or elect not to answer any question. The participants received no compensation. On average, it took students 30 min to complete the questionnaires.

Of the 3017 adolescents who took part in total, the average participation rate in the four waves was 83%: November 2017 (Wave 1) (*n* = 2790, 92% participation rate, 49% girls; *M*_age_ = 13.15, *SD* = 1.09); May 2018 (W2) (*n* = 2553, 85% participation rate, 50% girls; *M*_age_ = 13.61, *SD* = 1.13); November 2018 (W3) (*n* = 2362, 78% participation rate, 51% girls; *M*_age_ = 14.03, *SD* = 1.05); and May 2019 (W4) (*n* = 2361, 78% participation rate, 50% girls; *M*_age_ = 14.55, *SD* = 1.06). In total, 1788 completed all four waves (59%, 51% girls), 675 completed three waves (22%, 47% girls), 336 participated in two waves (11%, 49% girls) and 214 students completed only a single wave (7%, 41% girls). Reasons for missing a wave included absence from school on the day of data collection and having left the school or having joined the study due to changing schools.

### Measures

#### Bullying perpetration

Bullying perpetration was measured with the subscale of the Spanish version of the European Bullying Intervention Project Questionnaire (EBIPQ) (Ortega-Ruiz et al., 2016). Example items on face-to-face overall perpetration include: “I have hit someone” and “I have excluded or ignored someone”. Participants were informed about distinguishing bullying from aggressive behaviors via the intentionality, repetition over time and the power imbalance in bullying prior to answering. The subscale comprises 7 items rated on a 5-point Likert-type scale, ranging from 0 (*Never*) to 4 (*More than once a week*). The final score is the average of all items, with high scores reflecting higher levels of bullying perpetration. The reliability of the Spanish version of the bullying subscale was α = 0.77 (Ortega-Ruiz et al., 2016).

#### Moral disengagement

In the Spanish version of the adolescent Moral Disengagement Scale (Caprara et al., 1995; Romera et al., 2021), participants rated 24 items about moral exoneration of negative behavior on a 5-point Likert-type scale, ranging from 1 (*Strongly disagree*) to 5 (*Strongly agree*). The reliability of the original scale was α = 0.85 (Caprara et al., 1995). The version used here included items from the adult version (Bandura et al., 1996) that were considered appropriate to measuring moral disengagement in an effective way in adolescents. The items focused on a variety of harmful behaviors and attitudes in daily contexts including: “teasing someone does not really hurt them” and “to hit obnoxious classmates is just teaching them a lesson”. Although originally the scale was aimed at assessing the multiple moral disengagement mechanisms, a one-factor structure has been widely used in previous studies using the mean score (Paciello et al., 2008).

#### Need for popularity

Behaviors reflecting strong motivation for social status and popularity was measured with the Popularity Scale (Santor et al., 2000), with participants rating items on the importance of achieving social-status goals and the efforts they have made to gain popularity amongst their peers. It comprises 12 items on a 7-point Likert-type scale, ranging from 1 (*Strongly disagree*) to 7 (*Strongly agree*). Examples of items are: “I’ve neglected some friends because of what other people might think” and “I have done things to make me more popular, even when it meant doing something I would not usually do”. Following the original conceptualization of the scale, the items were averaged to get an overall need for popularity score. Higher values indicate a greater need for popularity. The reliability of the original scale was α = 0.91. The reliability in the Spanish version was α = 0.93 and with acceptable psychometric properties (Del Rey et al., 2019).

#### Control variables

Gender (1 = *Boy*; 2 = *Girl*) and age at W1 were addressed as control variables.

### Statistical Analyses

Both cross-lagged panel models (CLPM) and random intercept cross-lagged panel models (RI-CLPM) were tested to explore the associations between the study variables over time. First, the CLPM was performed to explore the association between need for popularity, moral disengagement and bullying perpetration at the between-person level. The CLPM comprised stability paths (i.e., relative to other participants via autocorrelation), cross-lagged paths and cross-sectional covariances between the variables at the same time (from W2 to W4 the associations are focused on the residual covariances). The longitudinal influence in the associations between variables are contrasted with the group mean at the between-level (i.e., adolescents with higher scores relative to the mean moral disengagement at T1 predicts relatively higher bullying perpetration, again relative to the sample mean, at T2). Thus, significant cross-lagged paths indicate relative rank of a predictor accounting for changes in the relative rank of another variable at a subsequent time.

Secondly, a RI-CLPM was performed. At the between-person level, the associations between variables are analyzed based on time-invariant individual differences between adolescents using the random intercept factors. At the within-person level, the model comprised the same paths that in the CLPM. In this case, the cross-lagged paths capture the change in each variable in an individual that can be predicted by his or her own deviations in other variables from the previous wave (i.e., changes in need for popularity predict subsequent deviations in bullying perpetration compared to the individual’s *own* levels). Gender and age were introduced as time-invariant predictors of the observed variables in both models (Hamaker et al., 2015).

As preliminary steps, correlations between variables were run, and independent *t*-tests were applied to analyze the gender and age differences. Cronbach’s alpha coefficients were calculated to analyze the internal consistency of the scales. The longitudinal measurement invariance of each scale was analyzed to test whether the construct remained constant across waves and thus whether associations between variables in the CLPM and the RI-CLPM would be reliable. This was analyzed using a confirmatory factor analysis (CFA) in which covariance between items or correlated errors were not allowed. Increasingly restrictive steps of measurement invariance from the baseline to more restrictive models were implemented: (a) Configural: estimated without restrictions, in which factor loadings and intercepts were freely estimated; (b) Metric: equal factor loadings; and (c) Scalar: equal item intercepts in addition to equal loadings. In the analyses, adolescents were clustered within classrooms. The intraclass correlations (ICCs) for need for popularity, moral disengagement and bullying perpetration were computed to identify the variance that could be explained by stable differences among at the between-person relative to the variance explained by within-person fluctuations.

Since collecting data was gathered spatially separated over a period of approximately six months, a series of models of CLPM and RI-CLPM were compared by constraining different parameters, based on the principle of parsimony. A simplified model is preferred, because higher degrees of freedom increase the probability of rejection (Kline, 2015), while remaining conceptually consistent. Where no significant differences were reported between the simple (more degrees of freedom) and the complex model (less degrees of freedom), the simplest model was selected. The process of building the models involved four steps. First, the model was freely estimated without restrictions. Second, the autoregressive paths were constrained to be equal over time. Third, the constraints of the cross-lagged paths were included. Fourth, the residual covariances between the variables at the same time (from W2 to W4) were constrained over time.

Due to a high kurtosis and skewness found in the distributions of some variables (see Table 1), the robust maximum likelihood (MLR) estimator was used to account for non-normality. To assess model goodness of fit, the root mean square error of approximation (RMSEA) and the comparative fit index (CFI) were used, with prerequisites for acceptable model fit set at RMSEA < 0.08, and CFI > 0.90 (Hu & Bentler, 1999). In the nested models, statistically significant differences were reported when two of the following three criteria were matched: Δχ^2^ at *p* < 0.05 (Satorra & Bentler, 2001), ∆CFI  ≥ 0.01 and ∆RMSEA ≥ 0.015 (Chen, 2007). Analyses were performed using the M*plus* 8.4 (Muthén & Muthén, 1998). The command “type = complex” was adopted for the analyses with the purpose of correcting the standard errors based on the classrooms as a variable cluster. The hierarchical data structure was due to adolescents grouped by classrooms. It implies that at the between-person level high levels are referred to as adolescents who are compared to the class group average.

**Table 1:** Correlations, Descriptive Statistics and Cronbach’s Alpha

	1	2	3	4	5	6	7	8	9	10	11	12
1. Need for popularity W1	**–**											
2. Need for popularity W2	0.57^***^	**–**										
3. Need for popularity W3	0.53^***^	0.62^***^	**–**									
4. Need for popularity W4	0.49^***^	0.57^***^	0.66^***^	**–**								
5. Moral disengagement W1	0.39^***^	0.24^***^	0.22^***^	0.18^***^	**–**							
6. Moral disengagement W2	0.30^***^	0.35^***^	0.21^***^	0.23^***^	0.58^***^	**–**						
7. Moral disengagement W3	0.31^***^	0.28^***^	0.32^***^	0.26^***^	0.52^***^	0.56^***^	**–**					
8. Moral disengagement W4	0.27^***^	0.28^***^	0.24^***^	0.33^***^	0.45^***^	0.55^***^	0.62^***^	**–**				
9. Bullying perpetration W1	0.41^***^	0.25^***^	0.22^***^	0.18^***^	0.48^***^	0.35^***^	0.32^***^	0.32^***^	**–**			
10. Bullying perpetration W2	0.30^***^	0.33^***^	0.20^***^	0.18^***^	0.29^***^	0.45^***^	0.31^***^	0.30^***^	0.46^***^	**–**		
11. Bullying perpetration W3	0.29^***^	0.19^***^	0.32^***^	0.26^***^	0.30^***^	0.27^***^	0.43^***^	0.30^***^	0.38^***^	0.42^***^	**–**	
12. Bullying perpetration W4	0.22^***^	0.19^***^	0.29^***^	0.33^***^	0.25^***^	0.26^***^	0.31^***^	0.40^***^	0.30^***^	0.32^***^	0.43^***^	**–**
*M*	1.90	1.93	1.86	1.88	1.64	1.59	1.54	1.53	0.26	0.28	0.20	0.21
*SD*	1.02	1.01	0.96	0.99	0.55	0.55	0.51	0.53	0.44	0.45	0.38	0.36
﻿Skewness	1.81	1.64	1.62	1.61	1.63	1.81	1.83	1.96	3.45	3.33	3.56	3.48
Kurtosis	3.56	2.99	2.83	2.65	3.53	4.24	4.88	5.19	16.52	15.96	16.67	19.27
Cronbach’s α	0.87	0.89	0.89	0.90	0.89	0.90	0.89	0.90	0.82	0.81	0.81	0.78

***p < 0.001

Little’s test (Little, 1988) of the study variables provided a significant result (*p* < 0.001) indicating that data were not missing completely at random (MCAR). The normed version of χ^2^, which may be used to adjust the sensitivity to sample size (Bollen, 1989), was low (χ^2^/*df* = 1.31), indicating that the data were likely missing at random (MAR). Given these results, the full information maximum likelihood (FIML) procedure was considered. FIML provides an unbiased and effective parametric estimate in longitudinal studies. Moreover, using all available data for each participant prevents the loss of statistical power and other issues associated with traditional methods such as listwise and pairwise exclusion (Enders, 2010).

## Results

### Preliminary Analyses

Correlation coefficients, descriptive statistics and Cronbach’s alpha are reported in Table 1. Associations between variables were stable over the study period as shown by the correlation between waves: need for popularity (0.49 ≤ *r* ≤ 0.66), moral disengagement (0.45 ≤ *r* ≤ 0.62), and bullying (0.30 ≤ *r* ≤ 0.46). A positive association was found between need for popularity and moral disengagement, both within (0.32 ≤ *r* ≤ 0.39) and across waves (0.18 ≤ *r* ≤ 0.31). The association between need for popularity and bullying was also positive within (0.32 ≤ *r* ≤ 0.41) and across waves (0.18 ≤ *r* ≤ 0.30), as was that between moral disengagement and bullying, again both within (0.40 ≤ *r* ≤ 0.48) and across waves (0.25 ≤ *r* ≤ 0.35). Cronbach’s alpha indicated the three scales had good reliability in each wave (see Table 1).

Gender and age differences between study variables were explored with independent *t*-test. Boys scored higher than girls on all three variables in all waves (see Table 2). Older participants (14–16 years old) had statistically higher scores than younger participants (11–13 years old) on moral disengagement (in all four waves), need for popularity and bullying (in two waves for each variable) (see Table 2). All differences had low effect sizes, as measured through Cohen’s *d*.

**Table 2:** Gender and age descriptive statistics

	Gender *M* (*SD*)				Age *M* (*SD*)			
	Boys	Girls	*t-*test	*d*	11–13 years	14–16 years	*t-*test	*d*
Need for popularity W1	1.99 (1.07)	1.80 (0.99)	4.91***	0.19	1.85 (1.01)	1.96 (1.03)	−2.76**	0.11
Need for popularity W2	1.99 (1.04)	1.88 (0.91)	2.69**	0.12	1.91 (1.03)	1.98 (0.97)	−1.50	–
Need for popularity W3	1.91 (1.01)	1.82 (0.96)	2.04*	0.09	1.80 (0.93)	1.98 (0.99)	−4.22***	0.19
Need for popularity W4	1.92 (1.01)	1.84 (0.67)	2.01*	0.09	1.87 (1.00)	1.89 (0.96)	−0.54	–
Moral disengagement W1	1.75 (0.61)	1.52 (0.44)	10.83***	0.44	1.58 (0.53)	1.72 (0.55)	−6.00***	0.25
Moral disengagement W2	1.70 (0.61)	1.49 (0.47)	9.25***	0.39	1.55 (0.54)	1.65 (0.55)	−4.04***	0.19
Moral disengagement W3	1.64 (0.57)	1.45 (0.43)	8.82***	0.38	1.50 (0.51)	1.61 (0.50)	−4.37***	0.21
Moral disengagement W4	1.64 (0.60)	1.43 (0.43)	9.11***	0.39	1.51 (0.52)	1.57 (0.51)	−2.46*	0.12
Bullying perpetration W1	0.33 (0.52)	0.19 (0.32)	8.27***	0.32	0.23 (0.42)	0.31 (0.46)	−4.53***	0.18
Bullying perpetration W2	0.34 (0.52)	0.23 (0.37)	6.10***	0.25	0.26 (0.45)	0.33 (0.46)	−3.30**	0.15
Bullying perpetration W3	0.26 (0.45)	0.15 (0.29)	6.69***	0.28	0.19 (0.39)	0.21 (0.36)	−1.15	–
Bullying perpetration W4	0.25 (0.41)	0.17 (0.30)	5.24***	0.22	0.20 (0.35)	0.23 (0.37)	−1.71	–

*p < 0.05; **p < 0.01; ***p < 0.001

Whether the constructs were invariant over time was explored by measurement invariance. The configural invariance model without restrictions showed good baseline model fit for all three variables (see Table 3). Next, with factor loadings constrained to be equal across waves to test for metric invariance, fit model remained good and unchanged for all variables, given that no more than two criteria were violated by comparing the nested models. Finally, for scalar invariance (strong invariance) with intercepts constrained across waves, no significant change in fit was found (see Table 3). Overall, testing for measurement invariance revealed an invariant structure across waves for each of the scales, making them suitable for examination of the longitudinal associations between the variables.

**Table 3:** Testing for longitudinal measurement invariance

Model tested	Model fit indices			Model comparison		
	χ² (*df*)	CFI	RMSEA [90% CI]	∆χ² (*df*)	∆CFI	∆RMSEA
Need for popularity						
Configural	1584.472 (1020)***	0.992	0.014 [0.012, 0.015]	–	–	–
Metric	1626.165 (1050)***	0.992	0.014 [0.012, 0.015]	165.824 (30)***	0.000	0.000
Scalar	1815.194 (1215)***	0.992	0.013 [0.012, 0.014]	289.043 (165)***	0.000	−0.001
Moral disengagement						
Configural	5708.196 (4318)***	0.977	0.010 [0.010, 0.011]	–	–	–
Metric	5780.256 (4384)***	0.977	0.010 [0.010, 0.011]	201.965 (66)***	0.000	0.000
Scalar	5984.136 (4596)***	0.977	0.010 [0.009, 0.011]	280.884 (212)**	0.000	0.000
Bullying perpetration						
Configural	550.613 (305)***	0.986	0.016 [0.014, 0.019]	–	–	–
Metric	560.394 (320)***	0.987	0.016 [0.014, 0.018]	28.867 (15)*	0.001	0.000
Scalar	596.223 (379)***	0.988	0.014 [0.012, 0.016]	76.479 (59)	0.001	−0.002

***p < 0.001

The ICCs indicate that 58, 58 and 43% of the variance of need for popularity, moral disengagement or bullying perpetration respectively could be explained by the between-person differences denoted by the differences between the classrooms, while 42, 42 and 57% by fluctuations within-person. This indicated the need for models sensitive to within-person variance, such as the RI-CLPM.

### Traditional Cross-Lagged Panel Modeling

To address the hypotheses of the present study at the between-person level, a standard CLPM was conducted to explore the association over time between need for popularity, moral disengagement and bullying perpetration. Model 1 without unconstrained paths had an excellent fit (see Table 4). In model 2, the autoregressive paths were constrained to be equal over time (∆χ² (6) = 17.13, *p* < 0.01, ∆CFI = −0.003 and ∆RMSEA = −0.003). Compared to model 1, model 2 was not significantly worse as two of the three criteria did not match. After the constraint of equality over waves of the cross-lagged paths were added in model 3, non-significant differences compared to model 2 were found (∆χ² (12) = 23.25, *p* < 0.05, ∆CFI = −0.004 and ∆RMSEA = −0.005). Finally, in model 4 the residual covariances between variables within the same wave (from W2 to W4) were not allowed to vary across time. Again, this did not affect model fit compared to model 3 (∆χ² (6) = 7.17, *p* > 0.05, ∆CFI = 0.000 and ∆RMSEA = −0.003). Given the lack of significant differences in model fit in CLPM, model 4, the most parsimonious model, was used to assess the associations between variables.

**Table 4:** Hierarchical cross-lagged panel model and random intercept cross-lagged panel model

	Cross-lagged panel model			Random intercept cross-lagged panel model		
Model tested	χ² (*df*)	CFI	RMSEA [90% CI]	χ² (*df*)	CFI	RMSEA [90% CI]
Model 1	358.581 (33)***	0.948	0.060 [0.054, 0.065]	115.534 (27)***	0.986	0.034 [0.028, 0.041]
Model 2	384.254 (39)***	0.945	0.057 [0.052, 0.062]	152.797 (33)***	0.981	0.036 [0.041, 0.042]
Model 3	425.270 (51)***	0.941	0.052 [0.047, 0.056]	184.260 (45)***	0.978	0.033 [0.029, 0.039]
Model 4	432.048 (57)***	0.941	0.049 [0.045, 0.053]	176.265 (51)***	0.980	0.030 [0.025, 0.035]

***p < 0.001

**Fig. 1:**
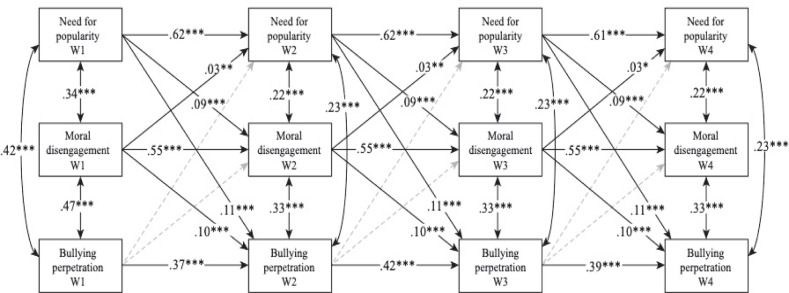
*Traditional Cross-Lagged Panel Model. Note:* The coefficients were standardized after estimation (model input was unstandardized). Dashed arrows show non-significant paths. ***p < 0.001

The associations between the variables from the between-person effects in the CLPM are illustrated in Fig. 1 through the standardized coefficients. The autoregressive paths were significant across waves (see Fig. 1). Both associations between the different variables (W1) and their residual covariances (from W2 to W4) within the same wave were all significant. Based on the hypotheses posed in the between-person level, the cross-lagged associations between the different variables showed that those adolescents with relatively higher need for popularity and moral disengagement (relative to the classmate average) at specific waves, reported relatively higher bullying than their peers at the subsequent wave, while the reverse influences were not found. In addition, bidirectional relationships were between need for popularity and moral disengagement at each wave, indicating that relatively high scores on need for popularity predicted relatively high scores on moral disengagement at the next wave, and vice versa. According to the effects of the time-invariant predictors, girls showed lower levels of involvement in all three of the focal study variables (see Table 5). Younger participants showed less need for popularity only in W3, less moral disengagement in W1, W2 and W3, and less bullying perpetration in W1 and W2 (see Table 5).

**Table 5:** Effects of time-invariant predictors on variables in CLPM and RI-CLPM

	CLPM				RI-CLPM			
	Gender		Age		Gender		Age	
	*b*	*SD*	*b*	*SD*	*b*	*SD*	*b*	*SD*
Need for popularity W1	−0.21***	0.01	0.06	0.04	−0.21***	0.01	0.06	0.04
Need for popularity W2	−0.15***	0.04	0.04	0.03	−0.15***	0.04	0.04	0.03
Need for popularity W3	−0.15***	0.04	0.08***	0.02	−0.14***	0.01	0.08*	0.02
Need for popularity W4	−0.12*	0.06	0.02	0.03	−0.13*	0.06	0.02	0.03
Moral disengagement W1	−0.25***	0.02	0.08***	0.02	−0.25***	0.02	0.07***	0.02
Moral disengagement W2	−0.24***	0.02	0.07***	0.02	−0.24***	0.02	0.07***	0.02
Moral disengagement W3	−0.22***	0.01	0.07***	0.01	−0.22***	0.00	0.07***	0.01
Moral disengagement W4	−0.22***	0.02	0.04	0.02	−0.22***	0.02	0.04	0.02
Bullying perpetration W1	−0.14***	0.00	0.04***	0.01	−0.14***	0.00	0.05***	0.01
Bullying perpetration W2	−0.13***	0.02	0.04**	0.02	−0.13***	0.02	0.04**	0.02
Bullying perpetration W3	−0.12***	0.02	0.01	0.01	−0.12***	0.02	0.02	0.02
Bullying perpetration W4	−0.09***	0.01	0.01	0.01	−0.10***	0.01	0.01	0.01

*p < 0.05; **p < 0.01; ***p < 0.001

### Random Intercept Cross-Lagged Panel Modeling

To address the hypotheses of the present study at the within-person level, a RI-CLPM was conducted to explore the association over time between need for popularity, moral disengagement and bullying perpetration. As in the CLPM, hierarchical models were estimated (see Table 4). Model 1 without unconstrained paths had an excellent fit. After the autoregressive paths was constrained in model 2, differences compared to model 1 were non-significant (∆χ² (6) = 45.83, *p* < 0.001, ∆CFI = −0.005 and ∆RMSEA = 0.002). In model 3, the cross-lagged paths were not allowed to vary across time, which did not affect model fit compared to model 2 (∆χ² (12) = 27.80, *p* < 0.01, ∆CFI = −0.003 and ∆RMSEA = −0.003). In model 4, the residual covariances between variables within the same wave (from W2 to W4) were constrained over time showing an excellent fit, and this did not differ significantly from the model 3 (∆χ² (6) = 1.86, *p* > 0.05, ∆CFI = 0.002 and ∆RMSEA = −0.003) . Consequently, the model 4 was retain as the most parsimonious model.

**Fig. 2:**
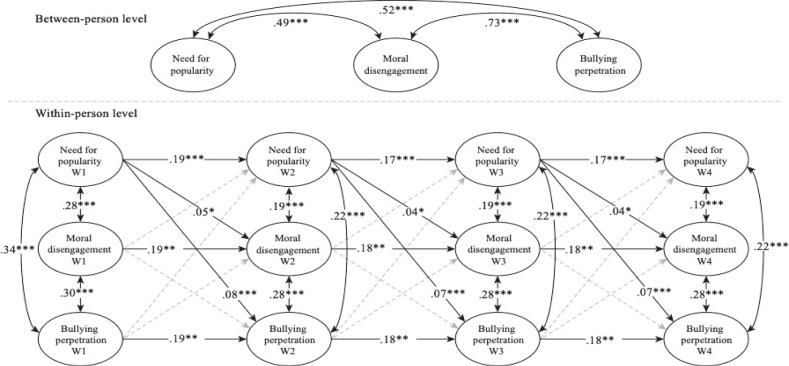
*Random Intercept Cross-Lagged Panel Model. Note:* The coefficients were standardized after estimation (model input was unstandardized). Dashed arrows show non-significant paths. *p < 0.05; **p < 0.01; ***p < 0.001

The associations between the variables are illustrated in Fig. 2 through the standardized coefficients. First, at the between-person level, the random intercept accounted for the stable differences between adolescents (i.e., with respect to their classmates) on the study variables. The random intercepts of need for popularity were positively correlated with moral disengagement and bullying, and the random intercepts of moral disengagement and bullying were also positively correlated (see Fig. 2). This suggests that those adolescents with high levels—compared with the average classroom—on one of the focal variables during any of the four waves also tended to have high levels on the other focal variables.

According to the study’s hypotheses at the within-person level, after accounting for the between-person variance, the autoregressive paths as well as the concurrent (within wave) correlations between the variables were all statistically significant. Furthermore, significant prospective associations highlighted that an increase in need for popularity yielded an increase in both moral disengagement and bullying perpetration in the subsequent wave, and this pattern held across all four waves (see Fig. 1). No significant cross-lagged was found in which moral disengagement and bullying perpetration were associated with subsequent waves of the other focal variables. After controlling for autoregressive effects and between-person variance, need for popularity predicted 4% of the explained within-person variance in moral disengagement six months later and between 4 and 5% in bullying perpetration. According to the effects of the time-invariant predictors, girls show a lower level of involvement in the study variables (see Table 5). Early adolescents showed less need for popularity only in W3, less moral disengagement in W1, W2 and W3, and less bullying perpetration in W1 and W2 (see Table 5).

### Sensitivity Analysis

Sensitive tests were conducted to guarantee the robustness of the results. Since the participants were clustered in schools, further analyses were performed taking into account this hierarchical structure. Through the command “type = complex” the analyses were re-run with the purpose of fixing the standard errors based on the school as a variable cluster. After obtaining a good fit index of the CLPM (χ² (57) = 266.327, *p* < 0.001, CFI = 0.948 and RMSEA = 0.036, 90% CI [0.032, 0.041]), the differences found (significant paths and size association between variables)—comparing when standard errors were controlled for classrooms—was that bullying perpetration predicted moral disengagement (*b* = 0.08, *SD* = 0.02, *p* < 0.001) and that need for popularity was not predicted by moral disengagement (*p* > 0.05). Regarding the RI-CLPM (χ² (51) = 104.182, *p* < 0.001, CFI = 0.987 and RMSEA = 0.019, 90% CI [0.014, 0.025]), after clustering among schools, differences were found in that changes in need for popularity did not produce subsequent deviations in moral disengagement (*p* > 0.05). Such results, however, should be taken with caution due to the low number of the clusters of the study (13 schools). Research has extensively discussed the minimum cluster size needed using hierarchical data structure; it has been consistently reported that with clusters smaller than 30, results should be treated cautiously as standard errors estimates may be biased (McNeish & Stapleton, 2016). Further information on the sensitivity analyses is available from the corresponding author on reasonable request.

## Discussion

The roles of motivational and moral factors in bullying have been considered independently and predominantly from a cross-sectional standpoint. The present study contributes to the literature on analyzing the association between need for popularity and moral disengagement. Furthermore, the present study used a statistical approach that accounts for methodological limitations in traditional cross-lagged panel modeling. Studying the longitudinal association between variables in cross-lagged paths from between- and within-level approaches contributes substantively to understanding the moral and motivational nature of perpetration during adolescence. The aim of the present study was to explore the prospective associations between moral disengagement, need for popularity and bullying perpetration in adolescents.

The proliferation of significant lagged-effects in data using traditional CLPM, where within-participant variance is not adequately accounted for, raises a serious question for prior studies: has evidence of lagged associations (Wang et al., 2017) been due to problematic analytic modeling of within-person variance? In the current study models, no support was found for the first hypothesis of bidirectionality over time between bullying perpetration and moral disengagement, once need for popularity was accounted for in the modeling. The traditional CLPM did, however, show a statistical influence of moral disengagement on subsequent bullying perpetration at each wave. The within-person findings suggest that deviations in moral disengagement and bullying perpetration were not significantly associated with changes within individuals on these variables. This suggests that between-person (time independent characteristics) differences may have been the primary determinant of significant longitudinal associations of previous studies (e.g., Visconti et al., 2015; Wang et al., 2017). Even though moral disengagement implies a process and a disposition, from a methodological view, it has been mainly conceptualized as an increasingly stable trait over time (Paciello et al., 2008). This study contributes to apply random intercepts in CLPM to the research of bullying. Future studies could examine whether a longer time interval than six months would capture influences on the changes between moral disengagement and bullying perpetration at the within-person level, or whether this influence begin prior to the age studied here.

This study furthers understanding of the longitudinal associations between need for popularity and bullying, accounting for both between-person and within-participant levels. Consistent with hypothesis, findings suggest that when adolescents have higher need for popularity than their peers, subsequent higher levels of bullying perpetration were also found (between-level). The within-level findings suggest that, for individuals who themselves become more focused on the need for popularity over time, their personal involvement in bullying perpetration is likely to increase as well, regardless of their ‘rank’ amongst their peers on these variables. These findings provide important evidence for the precedence of motivational factors in bullying perpetration, indicating that need for popularity may drive repeated increases within adolescents in their bullying perpetration over the six-month intervals assessed. In contrast, bullying did not appear to drive increases in the need for popularity, when using analyses that account for between- and within-person level. The findings support that conclusion that bullying perpetration in adolescence may be largely a function of proactive, deliberate and intentional behavior serving as a means to an end (Hawley, 1999). In adolescence, when social status is a priority and scarce resource to get (Cillessen & Marks, 2011), this motive may be particularly potent in shaping bullying behavior. Adolescents may adopt the behavior of popular peers precisely because they aim to achieve the social status those peers hold. Popularity has been widely found to be associated with various antisocial behaviors at this age, when rebellious rule-breaking may attain visibility and prestige in the group, with the added perception of greater agency and independence (Veenstra et al., 2018). In fact, it has been evidenced the relation between popularity and bullying perpetration at these ages. According to the studies of popularity, adolescents may imitate aggressive behavior as a way to improve their social position into the group (Veenstra & Huitsing, 2021). The present findings add to prior studies that have suggested that popularity motives predict increases in aggression (Dumas et al., 2019), and that those such ‘agentic’ goals are important drivers of aggression in adolescence (Ojanen & Nostrand, 2014), after accounting for methodologic differences that could have resulted in spurious relationships in previous studies. The findings were based on four waves of data and the patterns of between- and within-person effects were consistent over time. Future research is needed that explores whether individuals who manage to increase their popularity, or conversely who fail to attain a greater social status, continue to use bullying and aggression and/or moral disengagement to achieve social status.

Similarly, support was found for the third hypothesis, that need for popularity would predict increases in moral disengagement over time at the between- and within-level. This finding is consistent with prior research into the effects of social goals on moral disengagement (Visconti et al., 2015). This finding is based on social cognitive theory that identifies moral disengagement as a social-cognitive orientation that is strongly affected by social motivations. Self-interested instrumental goal pursuit may play a strong role in driving moral decision making (Arsenio & Lemerise, 2004). However, little research to our knowledge has examined how social- and self-oriented motivations might influence the development of moral engagement. Further research on such “motivated moral decision making” is needed to deepen the understanding of the relationship between popularity motivations and morality. Further research is needed, sensitive to development, to examine how motivations to achieve prestige and power within the peer group might serve as antecedents of moral disengagement.

### Limitations and Future Lines of Research

One limitation of the current study is its reliance on adolescents’ self-reports, which may have influenced findings through social desirability bias, acceptance bias, and participants’ mood. Adding reports from peers and teachers and other techniques (e.g., peer nomination) in future multi-level perspective studies could alleviate this weakness. The study also did not account for social status within the group, as sociometric assessment would enable. Given that some studies have found popularity motivations were not a significant when controlling actual popularity (Malamut et al., 2020) and others finding a significant interaction between popularity motivations and actual popularity (Dawes & Xie, 2014), such research is needed to clarify the picture. As well, need for popularity was the only motivational variable assessed; other motivations may drive moral disengagement and/or bullying, including revenge motives and recreational motives in bullying (Runions et al., 2018). As well, the bullying measure averaged over four modes of aggression (physical, indirect, relational, and social exclusion). Future studies can address this gap by testing motivational and moral precursors of these different types of bullying perpetration. Although the study used a large sample of adolescents, the results should be interpreted with care, as it consisted of schools from a single Spanish region that were not selected randomly. Stratified random sampling that includes adolescents from different cultural backgrounds would increase confidence in the results’ generality (Skrzypiec et al., 2018). Future studies should recruit adolescents belonging to minorities and marginalized groups. Regarding the methodology, whereas the RI-CLPM may capture changes from one time to the next and how the constructs are influenced, such findings could be further supported by considering prolonged changes over time (longer interval between waves and longer developmental spans of adolescence). Future research could employ methods such as growth modeling to explore whether possible trends of change over time in need for popularity, moral disengagement, and bullying perpetration are influenced by any other variables.

### Practical Implications

The results of this study offer teachers and practitioners a better understanding of how their students’ motivational strategies drive moral disengagement and bullying behavior. The robust evidence for need for popularity provides important direction for intervention. Within-person associations help to identify possible increases in need for popularity that may lead some adolescents to become targets for intervention. During adolescence, interventions that might aim to reduce aggression through addressing the need for popularity should consider peer group dynamics carefully: it is the group that gives and takes away popularity and the possible social benefits associated with it (Romera et al., 2019). If aggression no longer enhances popularity, the view that humiliating a peer will achieve benefits no longer makes sense. Promoting prosocial developmental goals may improve interpersonal relationships and thereby student wellbeing. Moreover, findings support that the most of relationship between variables were found at a dispositional level. It suggests that preventive efforts to address maladaptive behaviors should particularly target children from earlier ages, to promote that they learn to engage in a disposition to reject immoral actions, striving for aims of popularity and involvement in bullying behavior.

## Conclusion

Understanding what drives young people to engage in bullying is essential for effective prevention, especially for adolescents. Motivational and moral accounts of bullying perpetration have been studied extensively, but not often together and at both between- and within-person level. The present study differs from the research on the topic in developing an analytic approach that better accounts for the between- and within-person variance in predicting lagged associations over time. The RI-CLPM provides strong support for the role of need for popularity by replicating the traditional modeling. The findings of the study reveal potentially causal precedence of the need for popularity as a driver of bullying and moral disengagement. Popularity motivations changes appear to be addressed as a precursor of further changes in moral disengagement and perpetration. However, the findings suggest that the effects of moral disengagement on need for popularity and bullying perpetration are evident at between-level, but not at the within-person level. When moral disengagement is treated as a state, rather than as a stable characteristic, no influence is reported. When addressing moral disengagement as a process, this state approach seems not to be enough to capture a greater involvement in bullying six months later. The findings raise questions about the role of moral disengagement in the causal pathway to bullying in adolescence. This study opens a theoretical challenge in the study of the developmental importance of motivational mechanisms involved in bullying.

## References

[CR1] Arsenio WF, Lemerise EA (2004). Aggression and moral development: Integrating social information processing and moral domain models. Child Development.

[CR2] Bandura A (2018). A commentary on moral disengagement: the rhetoric and the reality. American Journal of Psychology.

[CR3] Bandura A (2002). Selective moral disengagement in the exercise of moral agency. Journal of Moral Education.

[CR4] Bandura A, Barbaranelli C, Caprara GV, Pastorelli C (1996). Mechanisms of moral disengagement in the exercise of moral agency. Journal of Personality and Social Psychology.

[CR6] Bollen, K. A. (1989). *Structural equations with latent variables*. Wiley. 10.1002/9781118619179

[CR7] Caprara GV, Barbaranelli C, Vicino S, Bandura A (1995). La misura del disimpegno morale (The assessment of moral disengagement). Rassegna Di Psicología.

[CR8] Cillessen, A. H. N., & Marks, P. E. L. (2011). Conceptualizing and measuring popularity. In A. H. N. Cillessen, D. Schwartz, & L. Mayeux (Eds.), *Popularity in the peer system* (pp. 25–56). The Guilford Press.

[CR9] Chen FF (2007). Sensitivity of goodness of fit indexes to lack of measurement invariance. Structural Equation Modeling.

[CR10] Cho S, Lee JR (2020). Joint growth trajectories of bullying perpetration and victimization among Korean adolescents: estimating a second-order growth mixture model–factor-of-curves with low self-control and opportunity correlates. Crime and Delinquency.

[CR11] Clark KN, Dorio NB, Demaray MK, Malecki CK (2020). Understanding bullying, victimization, and bystander behaviors through resource control theory. Child & Youth Care Forum.

[CR12] Dawes M, Xie H (2014). The role of popularity goal in early adolescents’ behaviors and popularity status. Developmental Psychology.

[CR13] Dawes M, Xie H (2017). The trajectory of popularity goal during the transition to middle school. Journal of Early Adolescence.

[CR14] Del Rey R, Ojeda M, Casas JA, Mora-Merchán JA, Elipe P (2019). Sexting among adolescents: The emotional impact and influence of the need for popularity. Frontiers in Psychology.

[CR70] Doty, J. L., Lynne, S. D., Metz, A. S., Yourell, J. L., & Espelage, D. L. (2020). Bullying Perpetration and Perceived Parental Monitoring: A Random Intercepts Cross-Lagged Panel Model. *Youth and Society*. 10.1177/0044118X20938416.

[CR15] Dumas TM, Davis JP, Ellis WE (2019). Is it good to be bad? A longitudinal analysis of adolescent popularity motivations as a predictor of engagement in relational aggression and risk behaviors. Youth & Society.

[CR16] Enders, C. K. (2010). *Applied missing data analysis*. Guilford Press.

[CR17] Falla, D., Ortega-Ruiz, R., Runions, K., & Romera, E. M. (2020). Why do victims become perpetrators of peer bullying? Moral disengagement in the cycle of violence. *Youth & Society*. 10.1177/0044118X20973702

[CR18] Galinsky AD, Magee JC, Ena Inesi M, Gruenfeld DH (2006). Power and perspectives not taken. Psychological Science.

[CR19] Gini G, Pozzoli T, Hymel S (2014). Moral disengagement among children and youth: a meta-analytic review of links to aggressive behavior. Aggressive Behavior.

[CR20] Hamaker EL, Kuiper RM, Grasman RP (2015). A critique of the cross-lagged panel model. Psychological Methods.

[CR21] Hawley PH (1999). The ontogenesis of social dominance: A strategy-based evolutionary perspective. Developmental Review.

[CR22] Hinrichs KT, Wang L, Hinrichs AT, Romero EJ (2012). Moral disengagement through displacement of responsibility: The role of leadership beliefs. Journal of Applied Social Psychology.

[CR23] Hogg, M. A. (2005). The social identity perspective. In *The handbook of group research and practice* (pp. 133–157). Sage. 10.4135/9781412990165.n8

[CR24] Hu LT, Bentler PM (1999). Cutoff criteria for fit indexes in covariance structure analysis: Conventional criteria versus new alternatives. Structural Equation Modeling.

[CR25] Hudson NW, Lucas RE, Donnellan MB (2019). Healthier and happier? A 3-year longitudinal investigation of the prospective associations and concurrent changes in health and experiential well-being. Personality and Social Psychology Bulletin.

[CR26] Huitsing G, Snijders TAB, Van Duijn MAJ, Veenstra R (2014). Victims, bullies, and their defenders: A longitudinal study of the coevolution of positive and negative networks. Development and Psychopathology.

[CR28] Kline, R. B. (2015). *Principles and practice of structural equation modeling*. Guilford Press.

[CR29] Little RJA (1988). A test of missing completely at random for multivariate data with missing values. Journal of the American Statistical Association.

[CR30] Makara KA, Madjar N (2015). The role of goal structures and peer climate in trajectories of social achievement goals during high school. Developmental Psychology.

[CR31] Malamut ST, van den Berg YHM, Lansu TAM, Cillessen AHN (2020). Bidirectional associations between popularity, popularity goal, and aggression, alcohol use and prosocial behaviors in adolescence: a 3-year prospective longitudinal study. ournal of Youth and Adolescence.

[CR32] McDonald KL, Asher SR, Bukowski WM, Laursen B, Rubin KH (2018). Peer acceptance, peer rejetion, and popularity. Handbook of peer interactions, relationships, and groups.

[CR33] McNeish DM, Stapleton LM (2016). The effect of small sample size on two-level model estimates: a review and illustration. Educational Psychology Review.

[CR34] Meisel, S. N., Paul, M. J., & Colder, C. R. (2021). Agency, communion, and pubertal status: Separating between- and within-person associations to examine social goals development. *Journal of Personality*. 10.1111/jopy.12638 10.1111/jopy.12638 PMC8487913 33835492

[CR35] Moffitt TE (1993). Adolescence-limited and life-course-persistent antisocial behavior: a developmental taxonomy. Psychological Review.

[CR36] Moore C (2015). Moral disengagement. Current Opinion in Psychology.

[CR37] Muthén, L. K., & Muthén, B. O. (1998). *Mplus user’s guide* (8th ed.). Muthén & Muthén.

[CR38] Ojanen, T., & Findley-Van Nostrand, D. (2020). Adolescent social goal development: Mean-level changes and prediction by self-esteem and narcissism. *Journal of Genetic Psychology*. 10.1080/00221325.2020.1792401 10.1080/00221325.2020.1792401 32693702

[CR39] Ojanen T, Nostrand DFVan (2014). Social goals, aggression, peer preference, and popularity: Longitudinal links during middle school. Developmental Psychology.

[CR40] Ortega-Ruiz R, Del Rey R, Casas JA (2016). Evaluar el bullying y el cyberbullying validación española del EBIP-Q y del ECIP-Q. Psicologia Educativa.

[CR41] Paciello M, Fida R, Tramontano C, Lupinetti C, Caprara GV (2008). Stability and change of moral disengagement and its on aggression and violence in late adolescence. Child Development.

[CR42] Pan B, Zhang L, Ji L, Garandeau CF, Salmivalli C, Zhang W (2020). Classroom status hierarchy moderates the association between social dominance goals and bullying behavior in middle childhood and early adolescence. Journal of Youth and Adolescence.

[CR43] Piff, P. K., Stancato, D. M., Cot̂eb́, S., Mendoza-Denton, R., & Keltner, D. (2012). Higher social class predicts increased unethical behavior. *Proceedings of the National Academy of Sciences of the United States of America*, 109(11), 4086–4091. 10.1073/pnas.1118373109 10.1073/pnas.1118373109 PMC3306667 22371585

[CR44] Pouwels JL, van Noorden THJ, Caravita SCS (2019). Defending victims of bullying in the classroom: The role of moral responsibility and social costs. Journal of Experimental Social Psychology.

[CR45] Prinstein, M. J. (2018). *Popular: The power of likability in a status-obsessed world*. Amazon.

[CR46] Romera EM, Casas JA, Gómez-Ortiz O, Ortega-Ruiz R (2019). Moral domain as a risk and protective factor against bullying. An integrating perspective review on the complexity of morality. Aggression and Violent Behavior.

[CR47] Romera EM, Ortega-Ruiz R, Runions K, Falla D (2021). Moral disengagement strategies in online and offline bullying. Psychosocial Intervention.

[CR48] Runions KC, Bak M (2015). Online moral disengagement, cyberbullying, and cyber-aggression. Cyberpsychology, Behavior, and Social Networking.

[CR49] Runions KC, Salmivalli C, Shaw T, Burns S, Cross D (2018). Beyond the reactive-proactive dichotomy: Rage, revenge, reward, and recreational aggression predict early high school bully and bully/victim status. Aggressive Behavior.

[CR50] Santor DA, Messervey D, Kusumakar V (2000). Measuring peer pressure, popularity, and conformity in adolescent boys and girls: Predicting school performance, sexual attitudes, and substance abuse. Journal of Youth and Adolescence.

[CR51] Satorra A, Bentler PM (2001). A scaled difference chi-square test statistic for moment structure analysis. Psychometrika.

[CR52] Schaefer, U., & Bouwmeester, O. (2020). Reconceptualizing moral disengagement as a process: transcending overly liberal and overly conservative practice in the field. *Journal of Business Ethics*. 10.1007/s10551-020-04520-6

[CR54] Skrzypiec G, Alinsug E, Nasiruddin UA, Andreou E, Brighi A, Didaskalou E, Guarinie A, Kang S, Kaurg K, Kwonh S, Ortega-Ruiz R, Romera EM, Roussi-Vergoud C, Sandhug D, Sikorskaj I, Wyraa M, Yangk C (2018). Self-reported harm of adolescent peer aggression in three world regions. Child Abuse & Neglect.

[CR55] Smith, P. K. (Ed.) (2019). *Making an impact on school bullying*. Routledge.

[CR56] Smith PK, López-Castro L, Robinson S, Görzig A (2019). Consistency of gender differences in bullying in cross-cultural surveys. Aggression and Violent Behavior.

[CR57] Stevens, G. W. J. M., Veldkamp, C., Harakeh, Z., & Laninga-Wijnen, L. (2020). Associations between ethnic minority status and popularity in adolescence: the role of ethnic classroom composition and aggression. *Journal of Youth and Adolescence*, *49*, 605–617. 10.1007/s10964-020-01200-6. 10.1007/s10964-020-01200-6 PMC7064451 32034631

[CR58] Sticca F, Perren S (2015). The chicken and the egg: Longitudinal associations between moral deficiencies and bullying: a parallel process latent growth model. Merrill-Palmer Quarterly.

[CR59] Teng Z, Nie Q, Guo C, Zhang Q, Liu Y, Bushman BJ (2019). A longitudinal study of link between exposure to violent video games and aggression in Chinese adolescents: the mediating role of moral disengagement. Developmental Psychology.

[CR61] Utz S, Tanis M, Vermeulen I (2012). It is all about being popular: The effects of need for popularity on social network site use. Cyberpsychology, Behavior, and Social Networking.

[CR63] Vanden Abeele M, Campbell SW, Eggermont S, Roe K (2014). Sexting, mobile porn use, and peer group dynamics: Boys’ and girls’ self-perceived popularity, need for popularity, and perceived peer pressure. Media Psychology.

[CR64] Veenstra R, Dijkstra JK, Kreager DA, Bukowski WM, Laursen B, Rubin KH (2018). Pathways, Networks, and Norms: A Sociological Perspective on Peer Research. Handbook of Peer Interactions, Relationships, and Groups.

[CR65] Veenstra, R., & Huitsing, G. (2021). Social network approaches to bullying and victimization. In P. K. Smith, & J. O’Higgins Norman (Eds.), *Handbook of Bullying. Volume 1: Characteristics, risks and outcomes* (pp. 191–210). Wiley-Blackwell.

[CR66] Visconti KJ, Ladd GW, Kochenderfer-Ladd B (2015). The role of moral disengagement in the associations between children’s social goals and aggression. Merrill-Palmer Quarterly.

[CR67] Wang C, Ryoo JH, Swearer SM, Turner R, Goldberg TS (2017). Longitudinal relationships between bullying and moral disengagement among adolescents. Journal of Youth and Adolescence.

[CR69] Zych I, Ttofi MM, Llorent VJ, Farrington DP, Ribeaud D, Eisner MP (2020). A longitudinal study on stability and transitions among bullying roles. Child Development.

